# Influenza symptoms and their impact on elderly adults: randomised trial of AS03-adjuvanted or non-adjuvanted inactivated trivalent seasonal influenza vaccines

**DOI:** 10.1111/irv.12245

**Published:** 2014-04-04

**Authors:** Gerrit A van Essen, Jiri Beran, Jeanne-Marie Devaster, Christelle Durand, Xavier Duval, Meral Esen, Ann R Falsey, Gregory Feldman, Pierre Gervais, Bruce L Innis, Martina Kovac, Odile Launay, Geert Leroux-Roels, Janet E McElhaney, Shelly McNeil, Mohammed Oujaa, Jan Hendrik Richardus, Guillermo Ruiz-Palacios, Richard H Osborne, Lidia Oostvogels

**Affiliations:** a Julius Center for Health Sciences and Primary Care, University Medical Center UtrechtUtrecht, The Netherlands; b Vaccination and Travel Medicine CentrePoliklinika 2, Czech Republic; c GlaxoSmithKline VaccinesWavre, Belgium; d Inserm CIC 007, U 738, Hôpital Bichat Claude BernardParis, France; e Institut für TropenmedizinTübingen, Germany; f University of Rochester Medical CenterRochester, NY, USA; g S. Carolina Pharmaceutical ResearchSpartanburg, SC, USA; h Q&T Research SherbrookeSherbrooke, QC, Canada; i GlaxoSmithKline VaccinesKing of Prussia, PA, USA; j Inserm, CIC BT505, and National Network of Clinical Investigation in Vaccinology (REIVAC)Paris, France; k Université Paris DescartesParis, France; l Assistance Publique Hôpitaux de Paris, Groupe Hospitalier, Paris Centre - CIC de Vaccinologie Cochin/PasteurParis, France; m Centre for Vaccinology, Ghent University and HospitalGhent, Belgium; n HSN Volunteer Association Chair in Geriatric Research, Advanced Medical Research Institute of CanadaSudbury, ON, Canada; o Queen Elizabeth Health Sciences Centre, Dalhousie University, PCIRN, NACI, CCfV, CAIRE, QEII HSC - VG Site Infectious DiseasesHalifax, NS, Canada; p GGD Rotterdam-RijnmondRotterdam, The Netherlands; q Department of Infectious Diseases, Instituto Nacional de Ciencias Médicas y Nutrición Salvador ZubiránMéxico City, México; r Public Health Innovation, Population Health Strategic Research Centre, Deakin UniversityMelbourne, Australia; s Measured Solutions for Health (MESH) P/LMelbourne, Australia

**Keywords:** AS03-adjuvanted vaccine, elderly, *FluiiQ*, influenza, patient-reported outcomes

## Abstract

**Background:**

Patient-reported outcomes (PROs) are particularly relevant in influenza vaccine trials in the elderly where reduction in symptom severity could prevent illness-related functional impairment.

**Objectives:**

To evaluate PROs in people aged ≥65 years receiving two different vaccines.

**Methods:**

This was a phase III, randomised, observer-blind study (NCT00753272) of the AS03-adjuvanted inactivated trivalent split-virion influenza vaccine (AS03-TIV) versus non-adjuvanted vaccine (TIV). Using the *FluiiQ* questionnaire, symptom (systemic, respiratory, total) and life impact (activities, emotions, relationships) scores were computed as exploratory endpoints, with minimal important difference (MID) in influenza severity between vaccines considered post-hoc as >7%. Vaccine efficacy of AS03-TIV relative to TIV in severe influenza (hospitalisation, complication, most severe one-third of episodes based on the area under the curve for systemic symptom score) was calculated post-hoc. The main analyses (descriptive) were conducted in the according-to-protocol cohort (*n* = 280 AS03-TIV, *n* = 315 TIV) for influenza confirmed by culture or reverse transcriptase polymerase chain reaction.

**Results:**

Mean systemic symptom, total symptom and impact on activities scores were lower with AS03-TIV versus TIV. Mean respiratory symptom, impact on emotions and impact on relationships scores were similar. Influenza tended to be less severe with AS03-TIV, but the MID was reached only for impact on activities (mean 9·0%). Relative vaccine efficacy in severe influenza was 29·38% (95% CI: 7·60–46·02).

**Conclusions:**

AS03-TIV had advantages over TIV in impact on systemic symptoms and activities as measured by the *FluiiQ* in elderly people. Higher efficacy of AS03-TIV relative to TIV was shown for prevention of severe illness.

## Introduction

The burden of hospitalisation and death in the elderly due to seasonal influenza is high,^1^–^3^ and vaccination against seasonal influenza is recommended routinely for individuals aged over 65 years in many countries. However, it is well recognised that the immune response to vaccination is lower in the elderly than in younger adults, with a corresponding decrease in vaccine effectiveness.^4^,^5^ One of the strategies to achieve a better immune response in the elderly is the use of adjuvants in vaccine formulations. The AS03 Adjuvant System is a tocopherol-based oil-in-water emulsion that has been approved for use in pandemic influenza vaccines in many countries. Studies have shown that vaccines formulated with AS03 are highly immunogenic against H5N1 avian strains and the human pandemic influenza H1N1 strain in elderly and non-elderly adults.^6^–^10^ A community-based case–control study of the MF59-adjuvanted trivalent seasonal vaccine has also shown benefit in the elderly versus a non-adjuvanted vaccine.^11^ Thus, adjuvanted vaccines provide a potential approach to reduce the incidence and severity of influenza in the elderly.

Patient-reported outcomes (PROs) have become recognised as important endpoints in clinical trials in many therapeutic areas, and guidelines for their development and use have been issued by the US Food and Drug Administration (FDA).^12^ However, clinical trials of influenza vaccines usually focus on prevention of infection. In the elderly, reduction in disease severity of breakthrough cases may be a more realistic goal and would represent a clinically significant achievement. Disease severity outcomes are best measured by PROs. A new instrument, the Influenza Intensity and Impact Questionnaire (*FluiiQ*), was recently developped to document PROs in influenza.^13^ The FDA has strict criteria governing the development and validation of PROs, and the *FluiiQ* is the only questionnaire developed for influenza to meet them. Development of the *FluiiQ* took place over three influenza seasons including data prospectively obtained from 25 sites across the USA.^13^

The present study evaluated a seasonal AS03-adjuvanted inactivated trivalent split-virion influenza vaccine (AS03-TIV) versus a non-adjuvanted trivalent vaccine (TIV) in an elderly population. Data from this study have been reported previously, showing that fewer participants receiving the AS03-TIV were infected with influenza A and/or B compared with TIV, although superiority of the AS03-TIV was not established.^14^ Because the primary efficacy endpoint of the study was not demonstrated, development of the AS03-TIV has been discontinued. Here, we describe PROs collected during the study.

## Methods

This was a phase III, randomised, observer-blind study (NCT00753272). Its main aim was to assess the relative efficacy of AS03-TIV versus TIV for the prevention of confirmed influenza A and B. The present paper focuses on PROs evaluated during the study. The trial was approved by relevant independent ethics committees or institutional review boards in each country, and was conducted in accordance with the Declaration of Helsinki, the International Conference on Harmonisation Good Clinical Practice guidelines, and regulatory requirements of participating countries. All participants provided written informed consent.

### Participants

Participants were recruited from Belgium, Canada, Czech Republic, Estonia, France, Germany, Mexico, Norway, Poland, Romania,^1^ Russia, Taiwan, the Netherlands, the United Kingdom and the USA and vaccinated during the usual Northern Hemisphere vaccination campaigns in the 2008/2009 and 2009/2010 influenza seasons. Because of the unexpected high circulation of the pandemic H1N1 strain during the 2009/2010 season and very few cases of seasonal influenza A and/or B, PROs were not analysed for the 2009/2010 season. Data are reported only for the 2008/2009 season. The first participant was vaccinated on 15 September 2008 and the last on 7 November 2008.

Participants were aged ≥65 years and lived in the community or in a retirement home that allowed mixing in the community. Participants who were in hospital, bedridden or who had an acute illness (moderate or severe illness with or without fever) were excluded. Other exclusion criteria included receipt of any influenza vaccine after February 2008 or vaccination in the previous 3 years with an investigational adjuvanted seasonal or pandemic influenza vaccine.

### Study design

Participants were randomised to receive AS03-TIV (0·7 ml) or TIV (0·5 ml; *Fluarix™*) vaccines, both manufactured by GlaxoSmithKline Vaccines. Both vaccines contained 15 μg haemagglutinin antigen per recommended influenza strain for the 2008/2009 season: A/Brisbane/59/2007 (H1N1), A/Uruguay/716/2007 (H3N2), B/Brisbane/3/2007 (B/Yamagata). The Adjuvant System contained 5·93 mg α-tocopherol and squalene in an oil-in-water emulsion (AS03_B_ formulation). A blocking scheme was used to randomise participants to the AS03-TIV and TIV groups with a 1:1 ratio. Randomisation was performed by the study sponsor and treatment allocation at each site was implemented using an internet-based system. Within each age stratum (65–74 years or ≥75 years), the randomisation algorithm used a minimisation procedure accounting for study centre and whether participants lived in a retirement home. Participants remained in the same treatment group in both influenza seasons. The vaccines were administered in the deltoid muscle of the non-dominant arm. Because they were slightly different in appearance, they were administered by personnel who took no further part in the study. Observers and participants were blind to vaccine allocation.

### Surveillance and identification of influenza-like illness and influenza

Active surveillance was performed from 15 November 2008 until the week of 30 April 2009, the most likely period of influenza virus circulation in the Northern Hemisphere. Participants were contacted biweekly, or weekly (between the weeks of 15 December and 31 March), and monitored for influenza-like illness (ILI), pneumonia, new onset or worsening congestive heart disease, myocardial infarction, stroke, respiratory disease causing hospitalisation, hospitalisation or urgent care visit, and serious or specified adverse events. In addition, participants were instructed to inform the study centre if they developed any of these events. ILI was defined as the simultaneous occurrence of at least one systemic symptom (headache, fatigue, myalgia, feverishness or fever [oral temperature ≥37·5°C]) and at least one respiratory symptom (nasal congestion, sore throat, cough, dyspnoea, sputum production or wheezing).

Nasal and throat swab specimens were collected within 5 days of the onset of ILI symptoms for confirmation of influenza virus A or B via reverse transcriptase polymerase chain reaction (RT-PCR) or via culture (Rhesus Monkey Kidney or Madin–Darby Canine Kidney tissue cultures) as previously described.^15^ The influenza peak season was defined for each participating country by an adjudication committee based on national surveillance and study data. Only cases with matching or drift influenza strains relative to the vaccine strains were included in the vaccine efficacy analysis, as a protocol amendment excluded the pandemic H1N1 strain from influenza-confirmed cases. However, the few pandemic H1N1 cases occurring during the 2008/2009 season were included in the PRO analysis.

### Study endpoints – patient-reported outcomes

Participants who experienced an ILI episode between 15 November 2008 to the end of the surveillance period and who provided a nasal or throat swab completed an ILI booklet that included the *FluiiQ*.^13^ The *FluiiQ* questionnaire was completed daily from the onset of the ILI episode until 15 days post-ILI onset. The *FluiiQ* consisted of five scales: systemic symptoms, respiratory symptoms, impact on daily activities, impact on emotions and impact on relationships. Each scale comprised 3–7 items with a 4-point Likert scale response option (0–3) where 0 indicates the absence of symptoms or absence of impact and 3 indicates highest severity of symptoms or highest impact.

### Statistics

All PROs were exploratory endpoints. Sample size calculations were based on the primary endpoint and are described elsewhere.^14^ SAS 9.2 was used for statistical analysis.

#### Analysis of PROs

Only ILI cases that occurred at least 2 weeks post-vaccination were included in the analysis to allow sufficient time for participants to mount an antibody response. Analysis of PROs was performed in the one-dose according-to-protocol (ATP) efficacy cohort which included all participants who received their first vaccine dose according to their random assignment, complied with the protocol and started their first active surveillance period (from 15 November 2008). In addition, participants who received their first vaccine dose after 1 November 2008 must not have discontinued the study within 2 weeks of vaccination to ensure at least 2 weeks of follow-up and sufficient time to mount an antibody response as stated above.

Within the one-dose ATP efficacy cohort, three subcohorts of participants who reported PROs were considered: (i) influenza-confirmed: all participants whose selected ILI episode was confirmed for influenza by PCR or culture and started within the surveillance period; (ii) ILI within peak season: all participants who experienced their selected ILI episode within the peak influenza season (both episodes confirmed and not confirmed for influenza by PCR or culture were included); (iii) influenza-negative: all participants whose selected ILI episode started outside the peak influenza season and was not confirmed for influenza by PCR or culture.

After computation of the *FluiiQ* scores, only one ILI episode per participant was considered in the analysis. If a participant experienced more than one influenza-confirmed ILI episode, the first episode was selected for the analysis. If a participant experienced several non-confirmed influenza ILI episodes, the episode included was selected as follows: (i) episode(s) for which a total symptom score was available for ≥5 days, with ≥1 day assessed during the first 5 days of the ILI; (ii) episode(s) that started during the peak influenza season; and (iii) one episode randomly selected. Only participants whose selected ILI episode had an evaluable PRO (≥5 days with an available total symptom score and ≥1 of these days assessed during the first 5 days of the ILI) were taken into account in the analysis.

#### Computation of *FluiiQ* scores

The *FluiiQ* was recorded daily. Each scale score was calculated by summing the responses to non-missing items recorded by individuals. The item response options ranged from 0 (no symptom/impact) to 3 (highest symptom intensity/impact). For each scale, the score was standardised to extend between 0 and 3 to be consistent with the response options and assist with interpretation of scores.

A score was considered as missing if the number of missing values on a particular scale was greater than or equal to half of the total number of items on a scale. Some missing scores were replaced by zero when at least one item on the scale had been recorded and the subject had indicated that they had ceased to complete the questionnaire because their symptoms had disappeared (applicable for all last days from which all items are equal to zero or missing and until Day 14) or had completed the questionnaire only on days when they experienced symptoms (applicable for all days from which all items are equal to zero or missing). Further imputation of missing values was a composite method based on (i) the last-observation-carried-forward method when zero was the last available score and (ii) multiple imputation using the Expectation-Maximisation algorithm (10 iterations of plausible values).^16^,^17^

Four derived variables based on each scale were calculated to evaluate the severity of clinical episodes: (i) maximum score throughout the 15-day follow-up period; (ii) area under the curve (AUC) from Day 0 to Day 14; (iii) AUC from Day 0 to Day 7; (iv) AUC from Day 0 to Day 4. The percentage difference between the TIV and AS03-TIV for the mean of the maximum score was calculated post-hoc as the absolute percentage change across the scale (0–3). In the absence of a gold standard in this field and to provide a benchmark for comparison, a minimal important difference (MID) was set at >7%.^18^–^20^ In addition, the time in days from Day 0 to total symptom score <0·5 was calculated to estimate the duration of clinical episodes.

Continuous parameters are presented using descriptive statistics (mean, standard error, minimum and maximum). No inferential analysis was performed. The analysis was based on multiple imputation, so 95% confidence intervals (CIs) were calculated according to the method of Rubin (procedure MIANALYZE).

#### Relative vaccine efficacy in severe illness

A post-hoc analysis evaluating severe influenza-confirmed episodes and severe ILI episodes was performed. Severe episodes were defined as those resulting in hospitalisation or a complication (death, pneumonia, myocardial infarction, stroke, congestive heart failure) or according to AUC (Day 0–Day 7) for the systemic and total symptom scores. For the AUC definition, one third of all episodes (those with the highest AUC) were considered to be severe. Episodes with no associated hospitalisation or complication, and with no or insufficient PRO information, were excluded from this analysis.

Vaccine efficacy of the AS03-TIV relative to the TIV in prevention of severe ILI and severe confirmed influenza A and/or B infection within the one-dose ATP efficacy cohort was calculated. Relative efficacy and 95% CIs were estimated by fitting a proportional hazards regression on the time-to-event, taking into account age (65–74 versus ≥75 years) and regional differences in attack rates (AR) (the Netherlands, Poland and Czech Republic [AR: 2·65–3·44%]; Belgium, UK, Norway, France, Germany and Russia [AR: 1·28–1·84%]; Canada, Romania, Mexico, Estonia, US and Taiwan [AR: 0·40–1·10%).

## Results

### Participants and compliance with ILI booklet completion

A total of 43 802 participants were enrolled, and 43 695 were vaccinated (21 893 and 21 802 in the AS03-TIV and TIV groups, respectively). A total of 664 and 718 participants in the AS03-TIV and TIV groups, respectively, withdrew from the study during the first year. The one-dose ATP efficacy cohort included 21 573 participants in the AS03-TIV group and 21 482 participants in the TIV group. Reasons for withdrawal and exclusion from the cohort are reported elsewhere.^14^ The mean age at first vaccination was 73·5 years in both vaccine groups, and 57% of participants in both groups were women. Most participants (86%) were of Caucasian origin.

Participants were included in the PRO analysis if they experienced an ILI and had an evaluable PRO for the selected episode. The number of participants in the AS03-TIV and TIV groups, respectively, for each subcohort was 280 and 315 (influenza-confirmed), 949 and 963 (ILI within peak season) and 960 and 982 (influenza-negative). Only one participant reported two influenza-confirmed episodes (one influenza A and the other influenza B), and only the first episode (influenza B) was taken into account in the analysis.

Compliance with completion of the ILI booklet was generally good, with similar percentages of participants completing at least 50% of the ILI booklet in both vaccine groups: 82·5% (AS03-TIV) and 83·3% (TIV) in the influenza-confirmed subcohort, 89·8% (AS03-TIV) and 91·1% (TIV) in the ILI within peak season subcohort, and 85·8% (AS03-TIV) and 82·7% (TIV) in the influenza-negative subcohort.

### Influenza symptom scores

**Figure 1:**
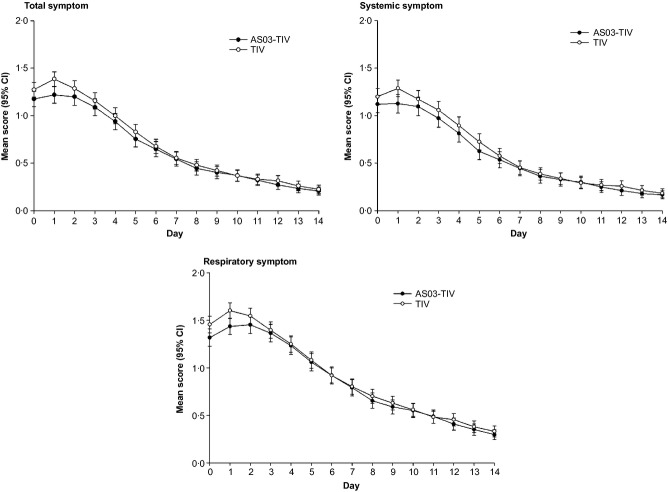
Total symptom, systemic symptom and respiratory symptom scores in the influenza-confirmed subcohort.

In the influenza-confirmed subcohort, the mean total symptom score and the mean systemic symptom score were lower in the AS03-TIV group than in the TIV group, particularly between Days 0 and 3, although the 95% CIs overlapped (Figure1). The mean difference between groups persisted for approximately 1 week. The respiratory symptom score was similar in both groups (Figure1). In the ILI within peak season subcohort and the influenza-negative subcohort, all symptom scores were similar in both vaccine groups (Figures S1 and S2). All three symptom scores were higher in the influenza-confirmed subcohort compared with the other two subcohorts (Figure1; Figures S1 and S2).

In all three subcohorts, the observed mean severity of ILI episodes was similar or less in the AS03-TIV group compared with the TIV group, based on the maximum score and AUC (Day 0–Day 14) for each symptom score (Table1). Differences in symptom scores between the AS03-TIV and the TIV were most marked in the influenza-confirmed subcohort; however, none reached the MID of 7% (Table1). Results were similar for severity based on AUC (Day 0–Day 4) and AUC (Day 0–Day 7), with similar or lower scores in the AS03-TIV group versus the TIV group (data not shown).

**Table 1:** Severity of ILI episode based on the mean of the maximum scores and the AUC from Day 0 to Day 14

Score	Subcohort								

	Confirmed influenza			ILI within peak season			Influenza-negative		
		
	AS03-TIV	TIV	Difference (%)	AS03-TIV	TIV	Difference (%)	AS03-TIV	TIV	Difference (%)
Total symptom									
Max score (SE)	1·50 (0·043)	1·64 (0·040)	4·7	1·33 (0·021)	1·38 (0·020)	1·7	1·32 (0·021)	1·36 (0·022)	1·3
AUC (SE)	9·12 (0·421)	9·81 (0·392)	–	7·76 (0·194)	8·10 (0·190)	–	8·00 (0·204)	8·16 (0·205)	–
Systemic symptom									
Max score (SE)	1·44 (0·049)	1·58 (0·047)	4·7	1·24 (0·023)	1·28 (0·023)	1·3	1·20 (0·023)	1·25 (0·024)	1·7
AUC (SE)	7·88 (0·447)	8·61 (0·420)	–	6·58 (0·199)	6·93 (0·198)	–	6·69 (0·206)	6·88 (0·211)	–
Respiratory symptom									
Max score (SE)	1·78 (0·045)	1·90 (0·040)	4·0	1·66 (0·023)	1·72 (0·022)	2·0	1·74 (0·023)	1·74 (0·023)	0·0
AUC (SE)	12·10 (0·446)	12·70 (0·416)	–	10·53 (0·231)	10·86 (0·219)	–	11·08 (0·241)	11·21 (0·238)	–
Impact on daily activities									
Max score (SE)	1·27 (0·065)	1·54 (0·062)	9·0	0·99 (0·030)	1·08 (0·031)	3·0	0·97 (0·030)	1·00 (0·030)	1·0
AUC (SE)	6·58 (0·520)	8·03 (0·513)	–	4·70 (0·206)	5·48 (0·232)	–	4·81 (0·212)	4·90 (0·223)	–
Impact on emotions									
Max score (SE)	0·89 (0·054)	1·02 (0·057)	4·3	0·75 (0·025)	0·83 (0·026)	2·7	0·77 (0·026)	0·82 (0·026)	1·7
AUC (SE)	4·54 (0·410)	5·39 (0·473)	–	3·78 (0·190)	4·23 (0·202)	–	3·89 (0·189)	4·10 (0·195)	–
Impact on relationships									
Max score (SE)	0·67 (0·050)	0·74 (0·051)	2·3	0·52 (0·022)	0·55 (0·023)	1·0	0·53 (0·022)	0·55 (0·023)	0·7
AUC (SE)	3·56 (0·404)	3·99 (0·419)	–	2·64 (0·164)	2·94 (0·174)	–	2·69 (0·161)	2·90 (0·171)	–

AUC: area under the curve; SE: standard error.

The percentage difference is calculated as the absolute percentage change across the scale (0–3): (TIV minus AS03-TIV) divided by 3*100. A difference of >7% was regarded as the MID.

The duration of ILI episodes using the total score tended to be shorter in the AS03-TIV group. In the AS03-TIV and TIV groups, respectively, the duration was 7·48 (standard error 0·287) and 7·84 (0·244) days in the influenza-confirmed subcohort, 6·40 (0·139) and 6·67 (0·137) days in the ILI within peak season subcohort, and 6·35 (0·140) and 6·36 (0·140) days in the influenza-negative subcohort.

There was no difference in severity and duration of ILI episodes in participants aged ≥75 years compared with those aged 65–74 years (data not shown). The vaccines had a similar impact on symptom severity in both age groups (data not shown).

### Impact on life scores

**Figure 2:**
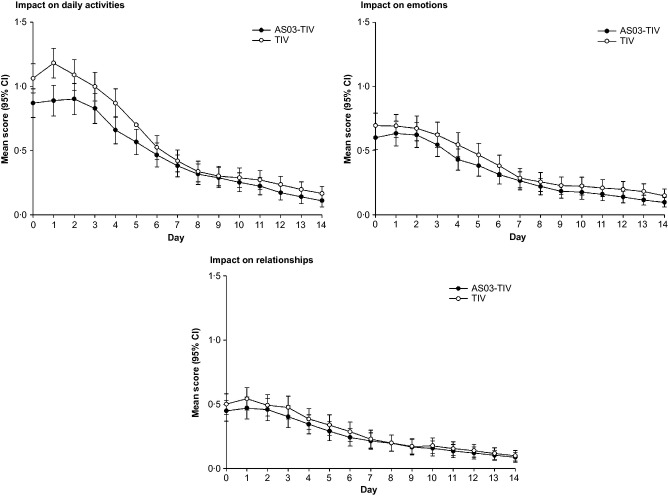
Impact on daily activities, emotions and relationships scores in the influenza-confirmed subcohort.

In the influenza-confirmed subcohort, the mean impact on daily activities score was lower in the AS03-TIV group, particularly during the first week (Figure2). However, the 95% CIs did not overlap only on Day 0 and Day 1. The impact on emotions score and impact on relationships score were similar in both vaccine groups (Figure2). As seen with symptom scores, life impact scores were similar in both vaccine groups in the ILI within peak season subcohort and the influenza-negative subcohort (Figures S3 and S4). All three life impact scores were higher in the influenza-confirmed subcohort compared with the ILI within peak season and influenza-negative subcohorts, particularly for the impact on daily activities score (Figure2; Figures S3 and S4).

The severity of ILI episode tended to be less in the AS03-TIV group compared with the TIV group. Again, differences were most marked in the influenza-confirmed subcohort; here, the difference between the AS03-TIV and TIV groups in severity based on the impact on daily activities score reached 9·0% (greater than the MID of 7%) (Table1).

The impact of influenza was greater for all scores in the ≥75 year age group than in the 65–74 year group; however, there was no difference between the vaccines when stratified by age (data not shown).

### Relative vaccine efficacy in severe illness

**Figure 3:**
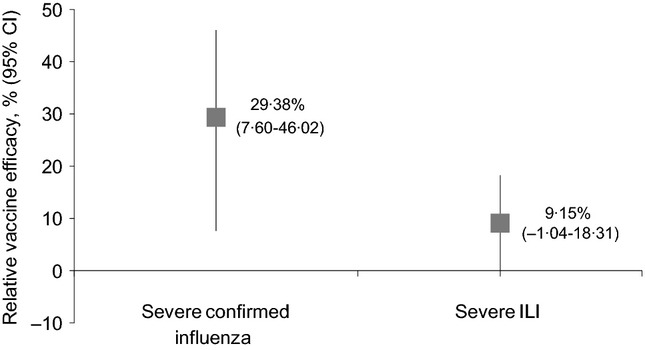
Vaccine efficacy of the AS03-TIV relative to the TIV for severe influenza-confirmed episodes and severe ILI episodes (based on systemic symptom score). Severe illness was defined as resulting in hospitalisation or complication (death, pneumonia, myocardial infarction, stroke, congestive heart failure), or illness with an AUC (Day 0–Day 7) for the systemic symptom score in the top one-third of all episodes.

A post-hoc analysis evaluated relative vaccine efficacy of the AS03-TIV compared with the TIV for prevention of severe influenza-confirmed and severe ILI episodes. There were 91 severe influenza-confirmed episodes in the AS03-TIV group compared with 128 episodes in the TIV group, with a relative vaccine efficacy of 29·38% (95% CI: 7·60–46·02) based on the definition using the systemic symptom score (Figure3). The corresponding relative vaccine efficacy in severe ILI was 9·15% (−1·04–18·31). Based on the definition using the total symptom score, relative vaccine efficacy was 29·03% (6·98–45·85) for severe influenza-confirmed episodes and 10·36% (0·33–19·39) for severe ILI episodes (Figure S5). Most severe influenza-confirmed episodes were caused by the A/H3N2 strain (76·7% in the AS03-TIV group and 84·1% in the TIV group).

## Discussion

To our knowledge, this is the first clinical efficacy trial of an influenza vaccine to report PROs due to the previous lack of a valid and reliable measure for influenza. Currently, complete prevention of influenza illness through vaccination appears to be unachievable, and thus, the primary aim of vaccination in elderly people may lie in the prevention of severe illness and reduction in morbidity and burden of influenza-associated complications. Such outcomes are ideally measured by PROs, which are relevant in the real-life setting and capture the experience of patients.

The efficacy of influenza vaccination is currently under question.^21^ Novel endpoints such as PROs and severe illness capture aspects of influenza that are relevant from both a clinical and public health perspective. It is therefore important that vaccine trials assessing potentially more efficacious influenza vaccines include such endpoints, as trials based solely on conventional endpoints might underestimate the value of vaccination. This may be particularly important in the elderly, because if an older person becomes bedbound or housebound for a period, they may lose function and independence which is never regained.^22^ Our analysis indicates that inclusion of PROs provides an additional dimension to clinical trials over and above traditional efficacy measures that are important to include in future studies.

The study showed a difference in favour of the AS03-TIV for the systemic symptom score and impact on daily activities score in the influenza-confirmed subcohort. The vaccines had a similar impact with respect to the respiratory symptom score, the impact on emotions score and the impact on relationships score. Likewise, the vaccines had a similar impact in the two other subcohorts evaluated, the ILI within peak season subcohort and the influenza-negative subcohort. Influenza was generally more severe in the TIV group than in the AS03-TIV group in all subcohorts and as measured by all symptom and impact on life scores. However, only the impact on daily activities score in the influenza-confirmed subcohort reached the benchmark MID of 7% between the TIV and AS03-TIV. The duration of ILI episodes tended to be shorter in the AS03-TIV group, although the difference between vaccines was not large.

The *FluiiQ* revealed the largest difference between the vaccines in the influenza-confirmed subcohort, which is expected as the *FluiiQ* was designed to be sensitive to influenza-specific outcomes,^13^ and the vaccine is effective exclusively against the influenza virus. By definition, all participants in the influenza-confirmed subcohort were infected with the influenza virus, whilst many participants in the other subcohorts could have had ILI caused by other viruses such as respiratory syncytial virus. It has been shown previously that the mean scores for severity of symptoms as measured by the *FluiiQ* questionnaire are higher in confirmed influenza compared with ILI, suggestive of more severe symptoms in the former.^13^

The post-hoc analysis of relative vaccine efficacy against severe influenza-confirmed episodes demonstrated a difference in efficacy in favour of the AS03-TIV. We chose to evaluate vaccine efficacy of the AS03-TIV relative to the TIV in severe confirmed influenza because of the importance of severe illness in older adults where the community burden associated with death and hospitalisation is high.^1^–^3^ We defined severe illness as a hospitalisation, complication (including death) or illness with an AUC for the systemic symptom score in the top one-third of all episodes. In an attempt to provide an overall perspective, we did the same analysis defining severe illness as a hospitalisation, complication or illness as measured by the *FluiiQ* total symptom score, and found similar results. It should be noted that calculation of a total score encompassing the systemic and respiratory items is not recommended because these have been found to be empirically different constructs, suggesting that they should not be summed.^13^ The finding in favour of the AS03-TIV in severe confirmed influenza gives another perspective in addition to the classical influenza-confirmed endpoint. Identification of vaccines that reduce severe influenza in the most vulnerable members of society is expected to have a positive impact on mortality and morbidity in these important target groups.

The possible mechanism for the findings in favour of the AS03-TIV must remain speculative, especially in view of the fact that analysis of the PROs was not inferential, the analysis of relative vaccine efficacy in severe illness was conducted post-hoc, and the primary efficacy endpoint of the study was not demonstrated. Higher antibody titres induced by the adjuvanted vaccine are likely to provide part of the explanation, but it should be noted that titres were measured only in subgroups of participants and therefore the antibody profile of all participants is unknown. It may also be possible that memory B-cell response may have been higher in participants receiving AS03-TIV, but this was not measured in the study. Cell-mediated immunity is likely to also play a role; indeed, T-cell responses have been shown to be better correlates of influenza vaccine protection in the elderly compared with antibody responses.^23^

The study had several strengths and limitations. A key strength was the use of the validated *FluiiQ* for measurement of PROs. Until now, influenza trials have been unable to study PROs comprehensively and reliably because of the lack of a validated measure to study the impact of influenza infection. The availability of the *FluiiQ*, a disease-specific measure of influenza, opens up the possibility to study the impact of vaccination on outcomes. In this study, we obtained a high response rate to the questionnaire in this elderly population, suggesting that the *FluiiQ* was easier to apply than perhaps initially believed. There are anecdotal reports from studies of anti-influenza treatments that the questionnaire actually helps to retain participants in a trial because they are routinely reminded of the trial through completing the questionnaire. The role of PROs in improving the quality of influenza research thus seems to be supported.

A limitation of our analysis of relative vaccine efficacy is that it is a post-hoc exploratory analysis not guided by specific hypotheses, therefore caution must be exercised in making conclusions about the efficacy of the AS03-TIV relative to the TIV in severe illness. Establishment of valid MIDs in PRO research remains problematic, and further studies are required to develop clinical benchmarks in support of what magnitudes of improvement in PROs are relevant in the influenza setting from both the clinical and public health perspectives.^18^ A further limitation is that the most severe cases might have been excluded from the analysis because they failed to complete the *FluiiQ*. In addition, we did not evaluate correlation of PROs with viral load. Strengths, limitations and generalisability of the trial as a whole have been previously described.^14^ The primary analysis illustrated a number of important lessons for the conduct of comparative vaccine trials in influenza.^14^

In conclusion, our exploratory analysis showed that the AS03-TIV has some advantages over the TIV in reduction of the impact of influenza on daily activities as measured by the *FluiiQ*, as well as in prevention of severe illness. Notwithstanding the caveats regarding efficacy, the results suggest that PRO evaluation and the impact of vaccination on disease severity and confirmed influenza should be the key components of future vaccine trials.

## Footnotes

1 There are concerns regarding the integrity of study data from a single study site in Romania which enrolled 102 subjects in the trial. At the time the concerns arose, the analyses for the study had already been completed. Because evaluation of data from this site did not reveal irregularities when compared with overall study data and because GSK has no current plans to use the data from the study in support of any regulatory filings, data from this site were not excluded from the analyses reported in this manuscript.

## Supporting Information

**Figure S1.** Total symptom, systemic symptom and respiratory symptom scores in the ILI within peak season subcohort.

**Figure S2.** Total symptom, systemic symptom and respiratory symptom scores in the influenza-negative subcohort.

**Figure S3.** Impact on daily activities, emotions and relationships scores in the ILI within peak season subcohort.

**Figure S4.** Impact on daily activities, emotions and relationships scores in the influenza-negative subcohort.

**Figure S5.** Vaccine efficacy of the AS03-TIV relative to the TIV for severe influenza-confirmed episodes and severe ILI episodes (based on total symptom score).
