# Supplementary figures and images for: Influenza symptoms and their impact on elderly adults: randomised trial of AS03-adjuvanted or non-adjuvanted inactivated trivalent seasonal influenza vaccines
Source: Influenza Other Respir Viruses. 2014 Apr 4;8(4):452–62. doi: 10.1111/irv.12245 (PMC4181805; doi:10.1111/irv.12245)

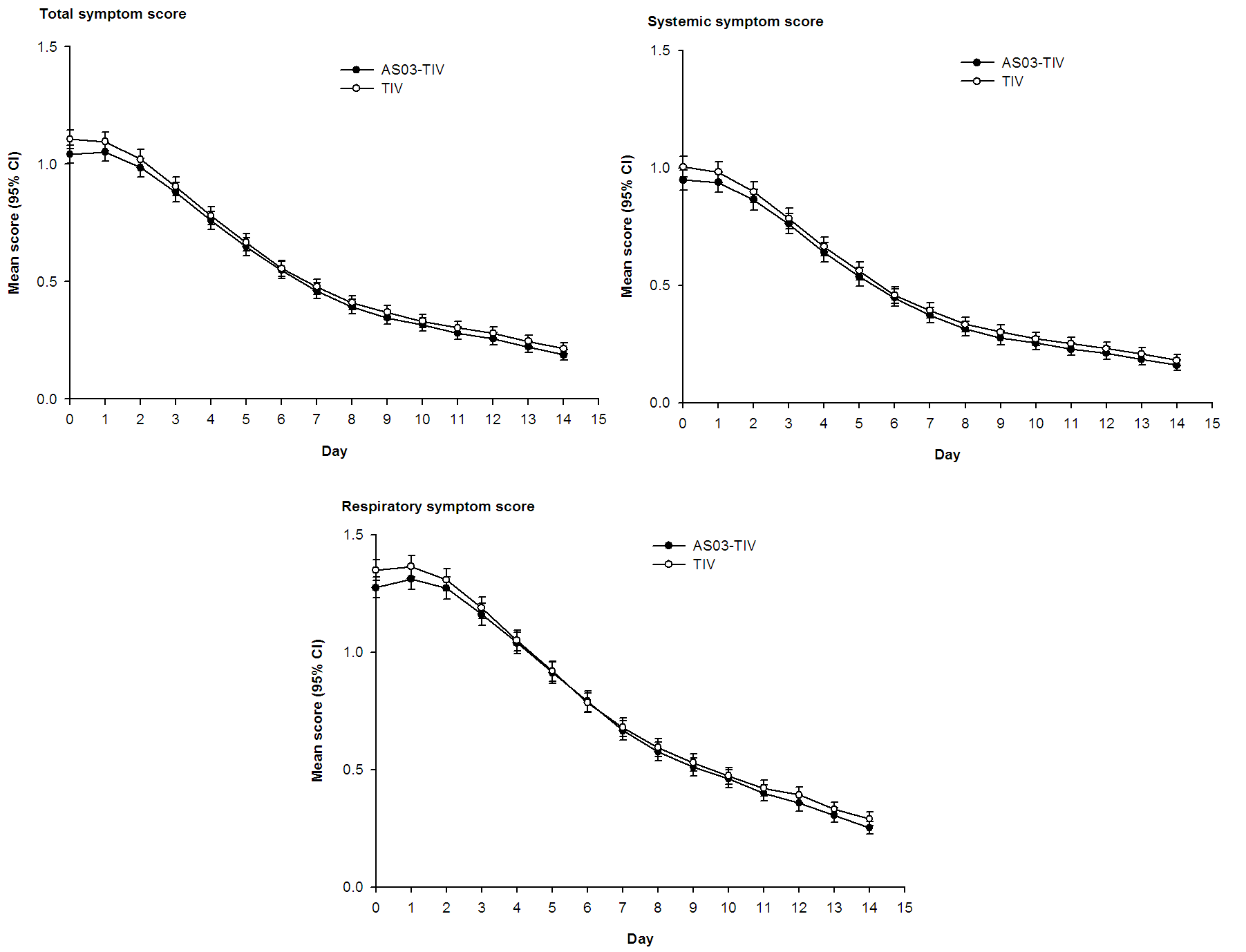

Supplement: Supplementary file 1 — Figure S1. Total symptom, systemic symptom and respiratory symptom scores in the ILI within peak season subcohort. [file irv0008-0452-SD1.tif]

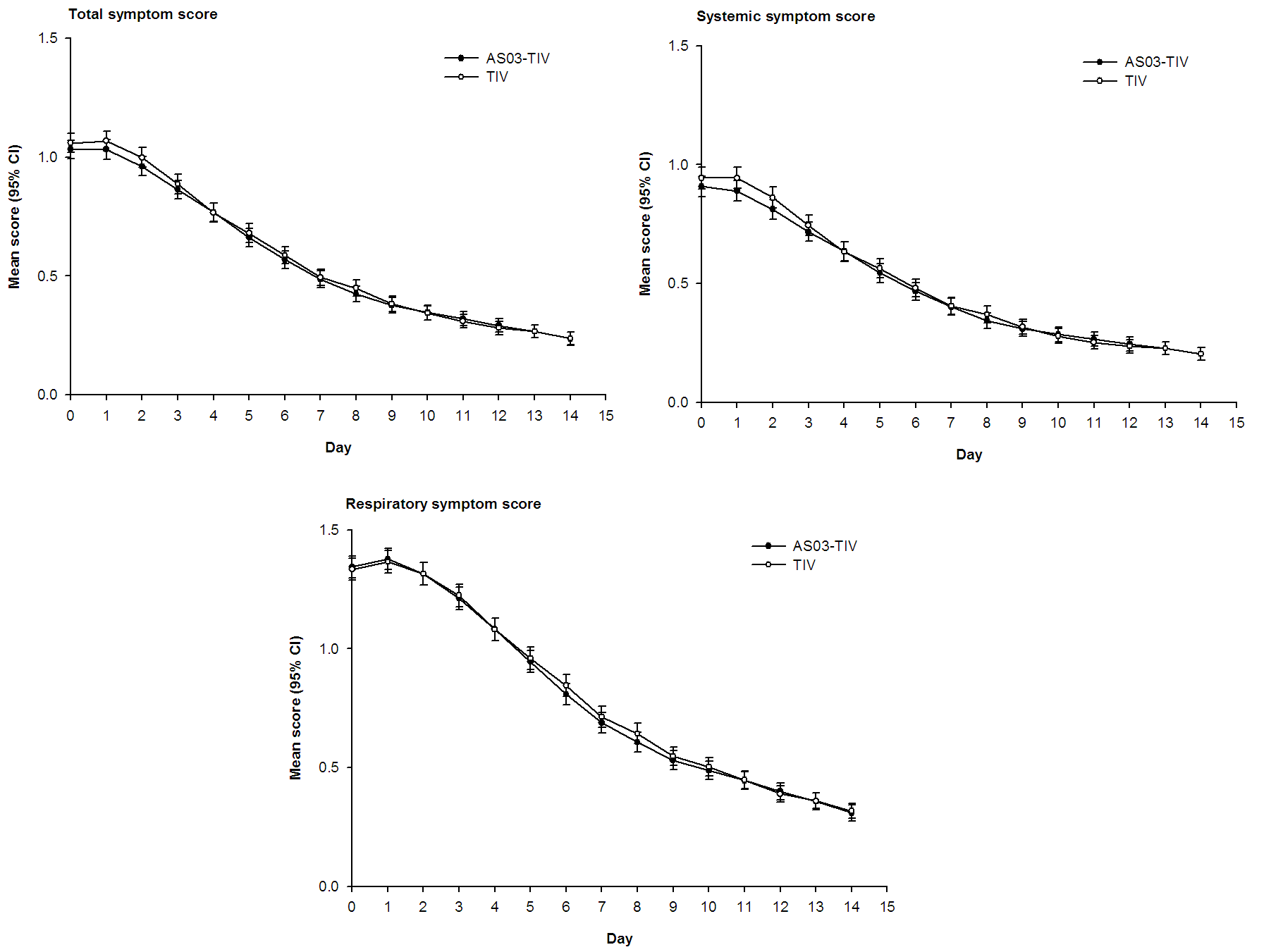

Supplement: Supplementary file 2 — Figure S2. Total symptom, systemic symptom and respiratory symptom scores in the influenza-negative subcohort. [file irv0008-0452-SD2.tif]

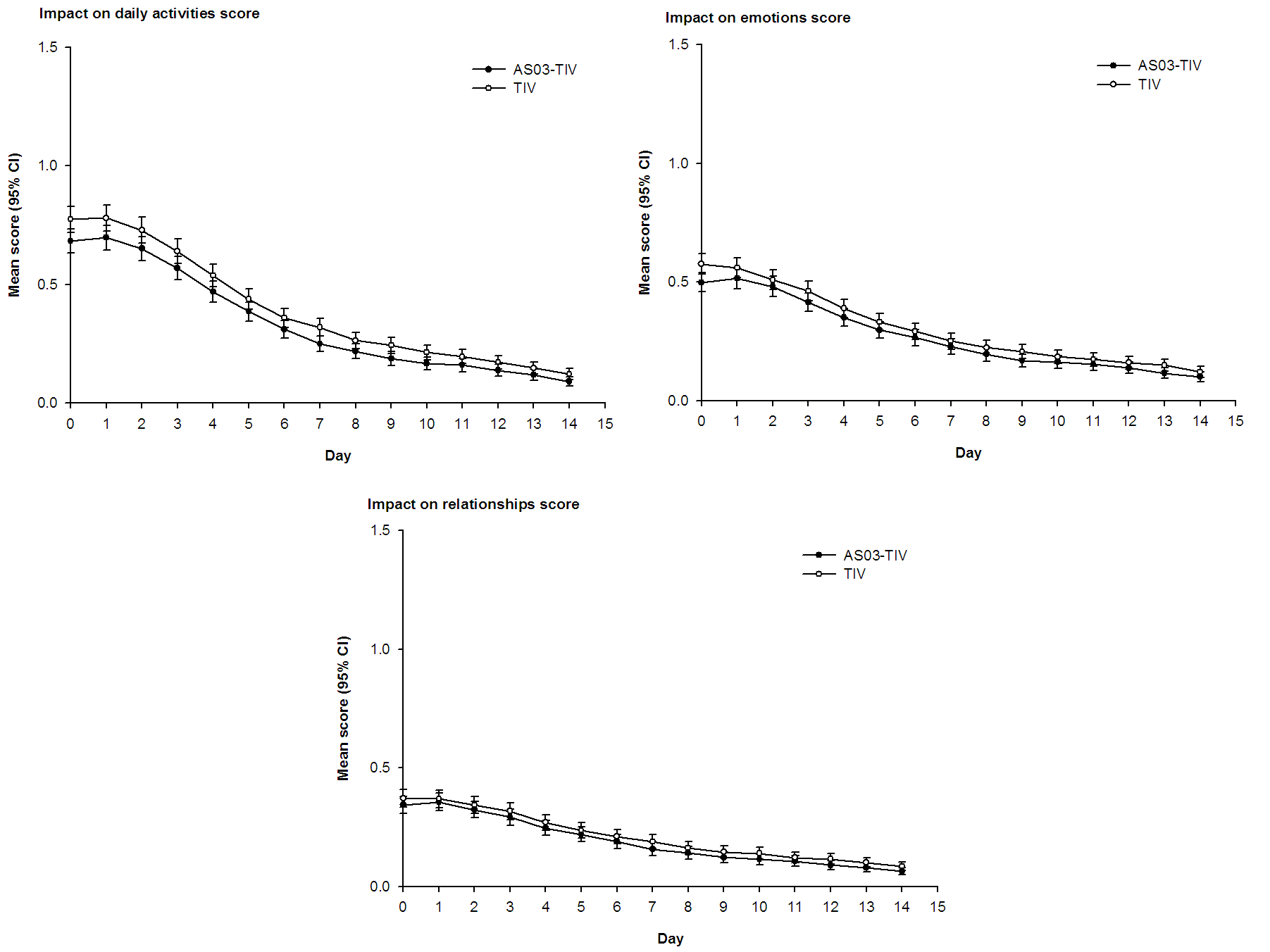

Supplement: Supplementary file 3 — Figure S3. Impact on daily activities, emotions and relationships scores in the ILI within peak season subcohort. [file irv0008-0452-SD3.tif]

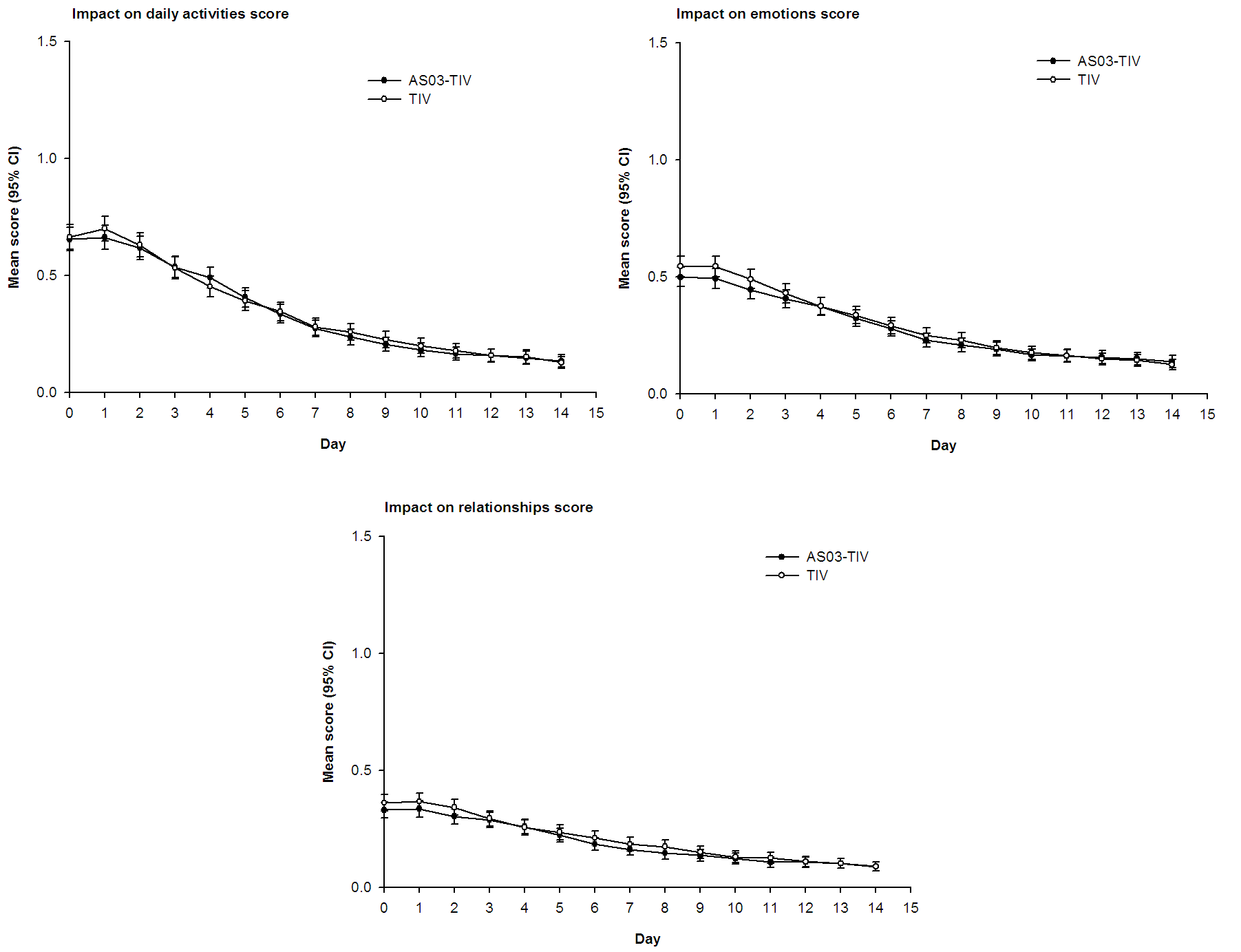

Supplement: Supplementary file 4 — Figure S4. Impact on daily activities, emotions and relationships scores in the influenza-negative subcohort. [file irv0008-0452-SD4.tif]

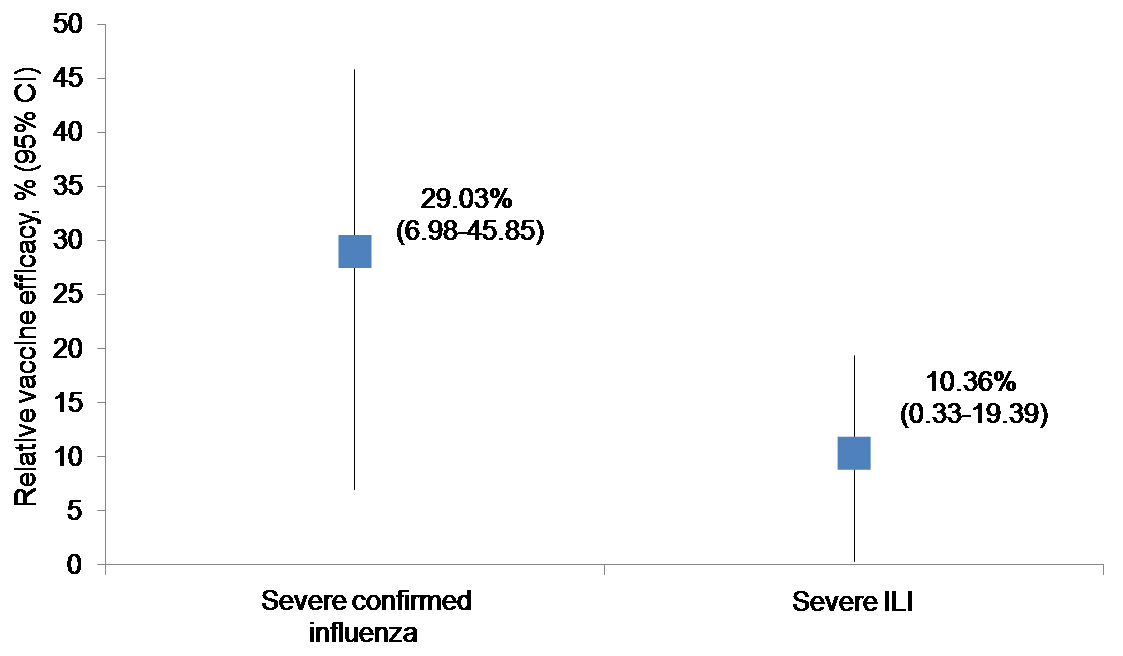

Supplement: Supplementary file 5 — Figure S5. Vaccine efficacy of the AS03-TIV relative to the TIV for severe influenza-confirmed episodes and severe ILI episodes (based on total symptom score). [file irv0008-0452-SD5.tif]
