# Supplementary material for: Influenza symptoms and their impact on elderly adults: randomised trial of AS03-adjuvanted or non-adjuvanted inactivated trivalent seasonal influenza vaccines
Source: Influenza Other Respir Viruses. 2014 Apr 4;8(4):452–62. doi: 10.1111/irv.12245 (PMC4181805; doi:10.1111/irv.12245)
Supplement: Supplementary file 6 — Figure S5. Vaccine efficacy of the AS03-TIV relative to the TIV for severe influenza-confirmed episodes and severe ILI episodes (based on total symptom score). [file irv0008-0452-SD6.docx]

Supplementary figure 1. Total symptom, systemic symptom and respiratory symptom scores in the ILI within peak season subcohort


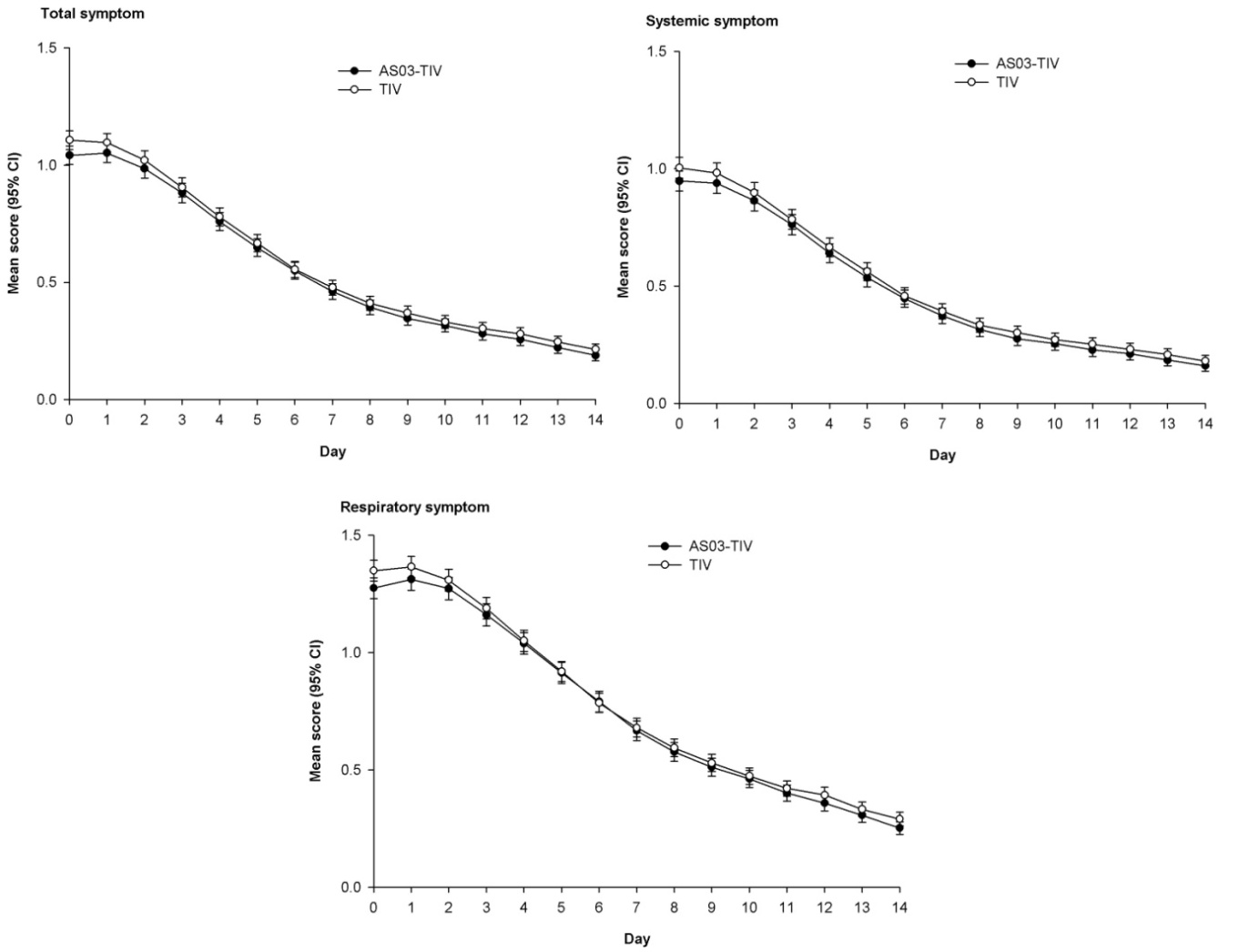


Supplementary figure 2. Total symptom, systemic symptom and respiratory symptom scores in the influenza-negative subcohort


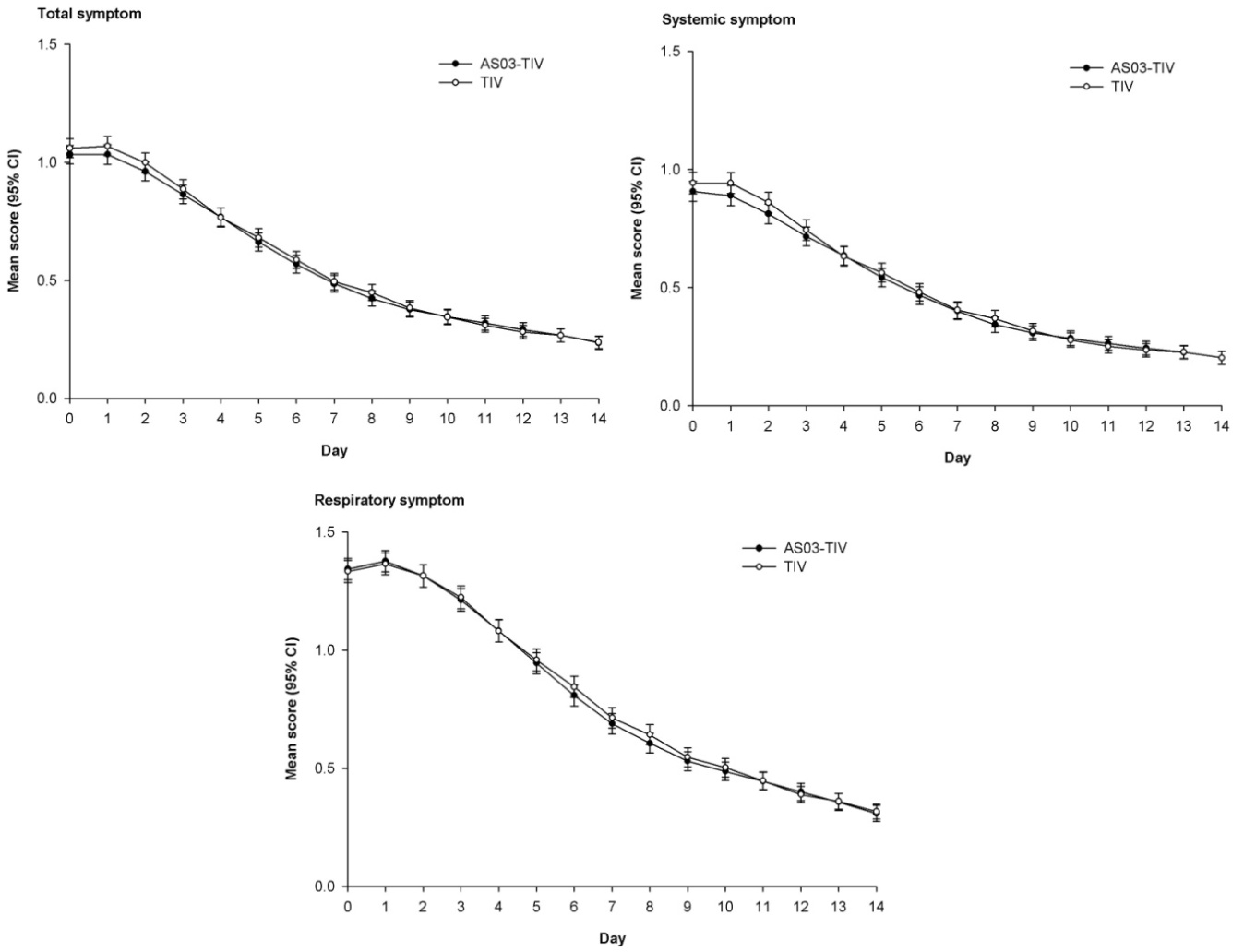


Supplementary figure 3. Impact on daily activities, emotions and relationships scores in the ILI within peak season subcohort


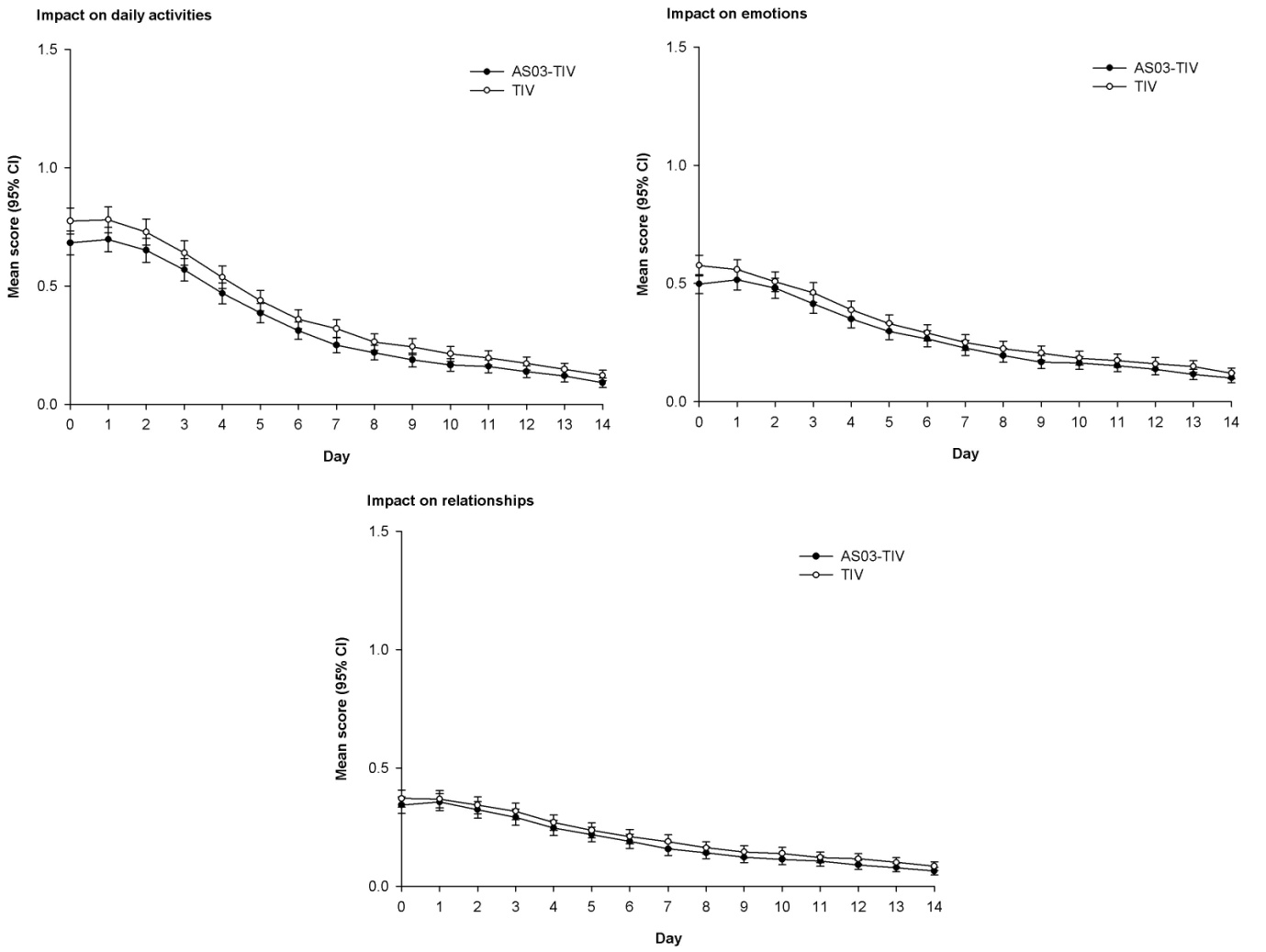


Supplementary figure 4. Impact on daily activities, emotions and relationships scores in the influenza-negative subcohort


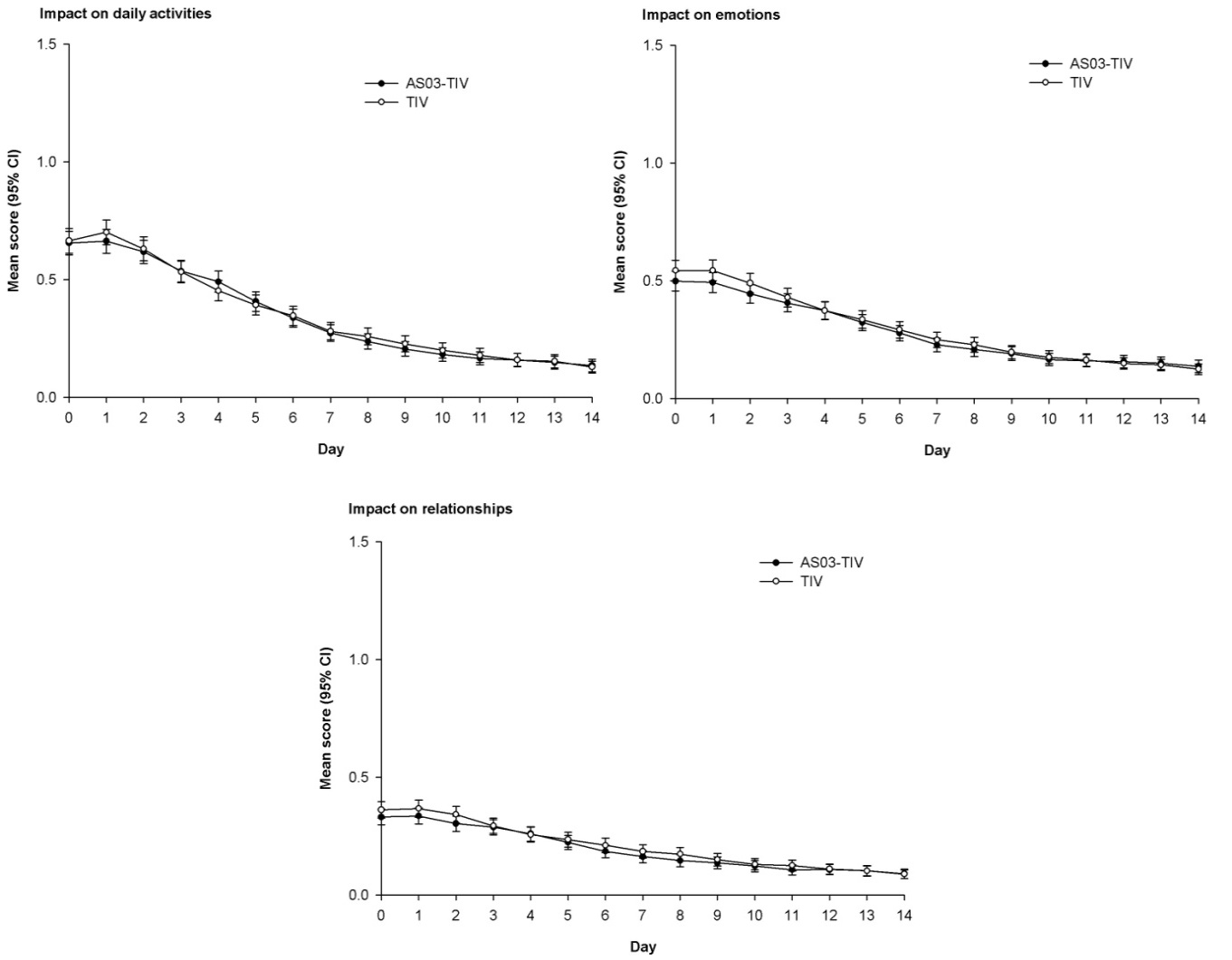


Supplementary figure 5. Vaccine efficacy of the AS03-TIV relative to the TIV for severe influenza-confirmed episodes and severe ILI episodes (based on total symptom score)

Severe illness was defined as resulting in hospitalisation or complication (death, pneumonia, myocardial infarction, stroke, congestive heart failure), or illness with an AUC (Day 0 to Day 7) for the total symptom score in the top one third of all episodes.
